# Lamination of primary visual cortex in the macaque: Layer 5 subdivisions

**DOI:** 10.1111/joa.70137

**Published:** 2026-04-09

**Authors:** Bashir Ahmed, Alvaro Duque, Pasko Rakic, Zoltán Molnár

**Affiliations:** ^1^ Department of Physiology, Anatomy and Genetics University of Oxford Oxford UK; ^2^ Department of Neuroscience Yale University School of Medicine New Haven Connecticut USA

**Keywords:** calcarine fissure, enucleated, histology, opercular region, QuPath: Open source software

## Abstract

Human and non‐human primates (NHPs) have a distinct lamination pattern of their primary visual cortex (V1) where layer 4 can be separated into four well‐defined sub‐laminae. Brodmann (1909) classified these into layers 4A, 4B, and 4C. Layer 4C can be further divided into 4Cα and 4Cβ based on inputs from the lateral geniculate magnocellular and parvocellular layers, respectively (Hubel & Wiesel, 1972; Lund, 1973). We have examined the lamination pattern of layer 5 and have found that this layer has a trilaminar structure and have denoted the sublayers as layers 5Aα, 5Aβ, and 5B. This trilaminar division is prominent within the opercular region of the occipital cortex but is usually absent from V1 surrounding the calcarine fissure. In addition, with age we find the trilaminar arrangement of layer 5 within the opercular region interdigitates with a bilaminar (denoted as layers 5Aβ, 5B) pattern. We also show that in a bilaterally enucleated macaque, at embryonic age of 63 days, postnatally, layer 5 is also trilaminar around the occipital cortex and bilaminar around the calcarine fissure. These facts imply that the interaction of the geniculo‐cortical input and local cortical development takes place in the absence of any visual experience, and we propose, functionally that this trilaminar arrangement of layer 5 may play a part in processing of central vision.

## Graphical abstract

We show a previously unrecognized trilaminar cytoarchitectonic organization within layer 5 of the opercular portion of the macaque primary visual cortex (V1), denoted as sub‐laminae 5Aα, 5Aβ, and 5B, a region which is involved in foveal and central visual field analyses. This trilaminar pattern is prominent during the first year postnatally but intermingles with a bilaminar arrangement in older macaques. We also show that within the calcarine fissure, a region of V1 responsible for peripheral and far peripheral vision, layer 5 is bilaminar. This pattern is also present in a macaque in which both eyes were removed in the embryo at a gestational age of 63 days, suggesting intrinsic developmental mechanisms. NeuN immuno‐labelled sections (A, B) are from macaque B65P78. Below A is tangential section from the opercular cortical region denoting the tri‐laminar arrangement of layer 5, and below B is tangential section from the calcarine region displaying the bilaminar arrangement of layer 5.

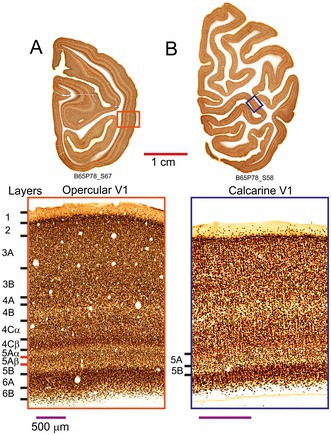

## 1 INTRODUCTION

**FIGURE 1:**
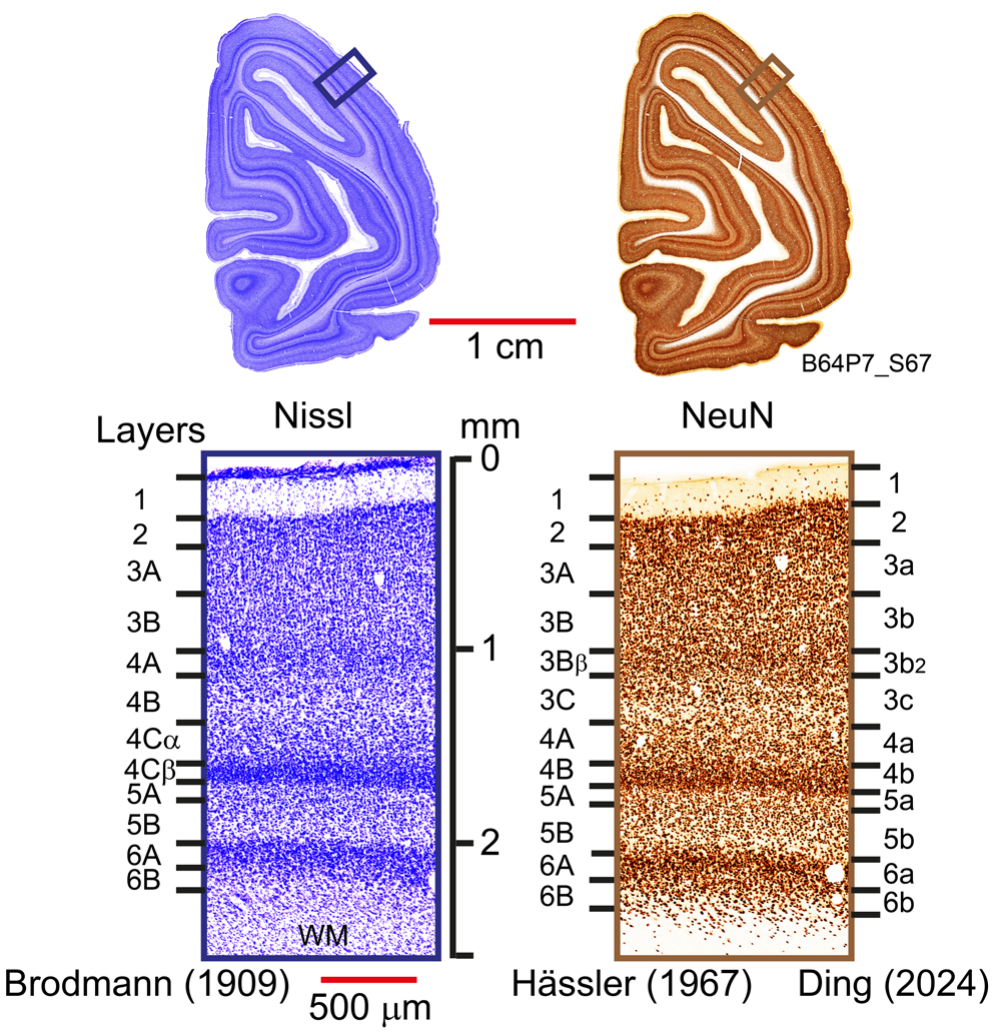
Coronal sections through occipital cortex labelled with Nissl and NeuN. Below the sections are shown the layering of the primary visual cortex (V1) at the denoted insets, respectively. The original layering schemes of Brodmann (1909) and Hässler (1967) used Roman numerals. Here, we have labelled them in Arabic numerals to make the comparison with Ding (2024). WM, white matter.

Primary visual cortex (V1) has a distinct layer pattern in humans and non‐human primates (NHPs) which is especially evident in layer 4. The original classification scheme of Brodmann (1909) subdivided layer 4 into three distinct compartments based on cell size and density: layers 4A, 4B, and 4C. Layer 4C itself is divided into two sublayers, layer 4Cα and layer 4Cβ, based on lateral geniculate input to these layers (Blasdel & Lund, 1983; Hendrickson et al., 1978; Hubel & Wiesel, 1972; Lund, 1973). Evidence that lateral geniculate input to layer 4B is sparse or absent (Blasdel & Lund, 1983; Fitzpatrick et al., 1983) together with layer 4B having projections to other cortical areas (Krubitzer & Kaas, 1990; Livingstone & Hubel, 1987; Shipp & Zeki, 1989) has led to a reclassification of layers 4A and 4B as part of layer 3 (Balaram & Kaas, 2014; Hässler, 1967). This has supported Hässler's laminar scheme where layers 4A and 4B were replaced with layers 3Bβ and 3C, respectively, and layers 4Cα and 4Cβ by layers 4A and 4B, respectively. Recently, Ding (2024) has put forward a scheme where the capital letters were replaced by lower case letters, to some extent to stop confusing Brodmann's layers 4A and 4B with Hässler's layers 4A and 4B. In order not to confound the situation, and as the extensive publications follow Broadmann's general scheme, in our paper we are continuing with this arrangement. Figure 1 summarises these laminar proposals.

Developmentally, the lamination pattern of V1 is completed in the embryo at gestation age of 112 days (Bourgeois & Rakic, 1993), and the developing neurons form not only accurate projections but also a high degree of selectivity to their main cortical laminae (Callaway, 1998). Further pruning of sub‐layer specific projections has been suggested to take place through prenatal activity in the visual network before birth (Callaway, 1998; Callaway & Wiser, 1996; Lund et al., 1977).

Layer 5 has historically been subdivided into two laminae, layers 5A and 5B. Layer 5A has been described as a narrow layer with a thickness of around 50 to 100 μm (Lund, 1987, 1988; Lund & Boothe, 1975) and densely packed compared with layer 5B (Balaram et al., 2014). The axonal arbours of the majority of neurons from layer 5 synapse within V1 (Callaway & Wiser, 1996; Briggs & Callaway, 2005) and a minority mainly in layer 5B, send axons subcortically (Lund & Boothe, 1975; Lund et al., 1988; Briggs & Callaway, 2005). The principal axonal projections of layer 5A are to layers 4C, 4A and to superficial laminae (Lund, 1987, 1988).

We have examined the lamination pattern of V1 in Nissl‐stained and NeuN immuno‐labelled material and have found that layer 5 can consistently be subdivided into three clearly defined laminae. These laminations are prominent around V1 of the opercular occipital cortex, representing central visual field, but less prominent or absent around the cortex overlying the calcarine fissure where V1 represents peripheral and far peripheral visual fields (Daniel & Whitteridge, 1961; Essen & Zeki, 1978; Zeki, 1969). In addition, we show that in the bilaterally enucleated macaque a similar pattern is present.

## 2 METHODS

This study is based on archived histological material which is publicly available from Collection 6 of the MacBrain Resource Centre hosted in the Department of Neuroscience at Yale University School of Medicine (https://medicine.yale.edu/neuroscience/macbrain/). Details of histological and immunostaining of tissue have been published (Duque et al., 2007, 2013; Rash et al., 2019).

We have examined high‐quality images obtained at ×20 of Nissl‐stained and NeuN immuno‐labelled coronal sections from 9 Macaques. The brains were sectioned in a 1:20 series, each section was 50 μm thick. Nissl labelled and NeuN immuno‐labelled sections were in the same series and at most 200 μm apart. Four were female macaques sacrificed at 7 and 77 days, 6.2 month, and 8.07 years postnatal (denoted, respectively, B64P7, B63P77, B89P186, and B72y8). Four were male macaques sacrificed at 75 and 78 days, 6 month, and 1.01 years postnatal (denoted, respectively, B61P75, B65P78, B80P180, and B87y1). One male macaque was sacrificed at an embryonic age of 143 days (B78E143). All these macaques had normal vision development. In addition, a Macaque who's left and right eyes were enucleated at embryonic age of 63 days and sacrificed at a postnatal age of 69 days (E63P69).

We have examined the laminar layout of layer 5 of V1 in coronal sections of the occipital cortex along the rostro‐caudal direction (i.e., from the opercular cortex to the calcarine fissure containing V1). The number of NeuN immuno‐labelled sections examined for each Macaque varied from 17 to 24, and for Nissl labelled sections from 19 to 25. Qualitatively, we have examined 179 and 196 coronal sections, respectively. For quantitative analyses, we picked representative examples of these coronal sections from the 8 Macaques at both the opercular (16 sections) and calcarine fissure (16 sections) regions. These coronal sections, analysed and quantified, were located within the primary visual cortex, ~ 4 mm–5 mm and ~10 mm–14 mm from the occipital pole. The former measurements were made at the opercular cortex and the latter measurements were made along the calcarine fissure.

The high‐quality images were examined by the use of the software QuPath (Bankhead et al., 2017, RRID:SCR_018257).

For each macaque, we present data from coronal sections at the level of the opercular region of the occipital cortex and from coronal sections at the level of occipital cortex where the calcarine fissure was prominent. Both these regions are primary visual cortex where the former represents central visual field and the latter peripheral and far peripheral visual fields (Daniel & Whitteridge, 1961; Essen & Zeki, 1978; Zeki, 1969). With the imaging software, we have measured thicknesses of cortex and thicknesses of the sub‐laminae of layer 5 at a range of positions along the coronal sections of both Nissl‐stained and NeuN immuno‐labelled sections.

## 3 RESULTS

**FIGURE 2:**
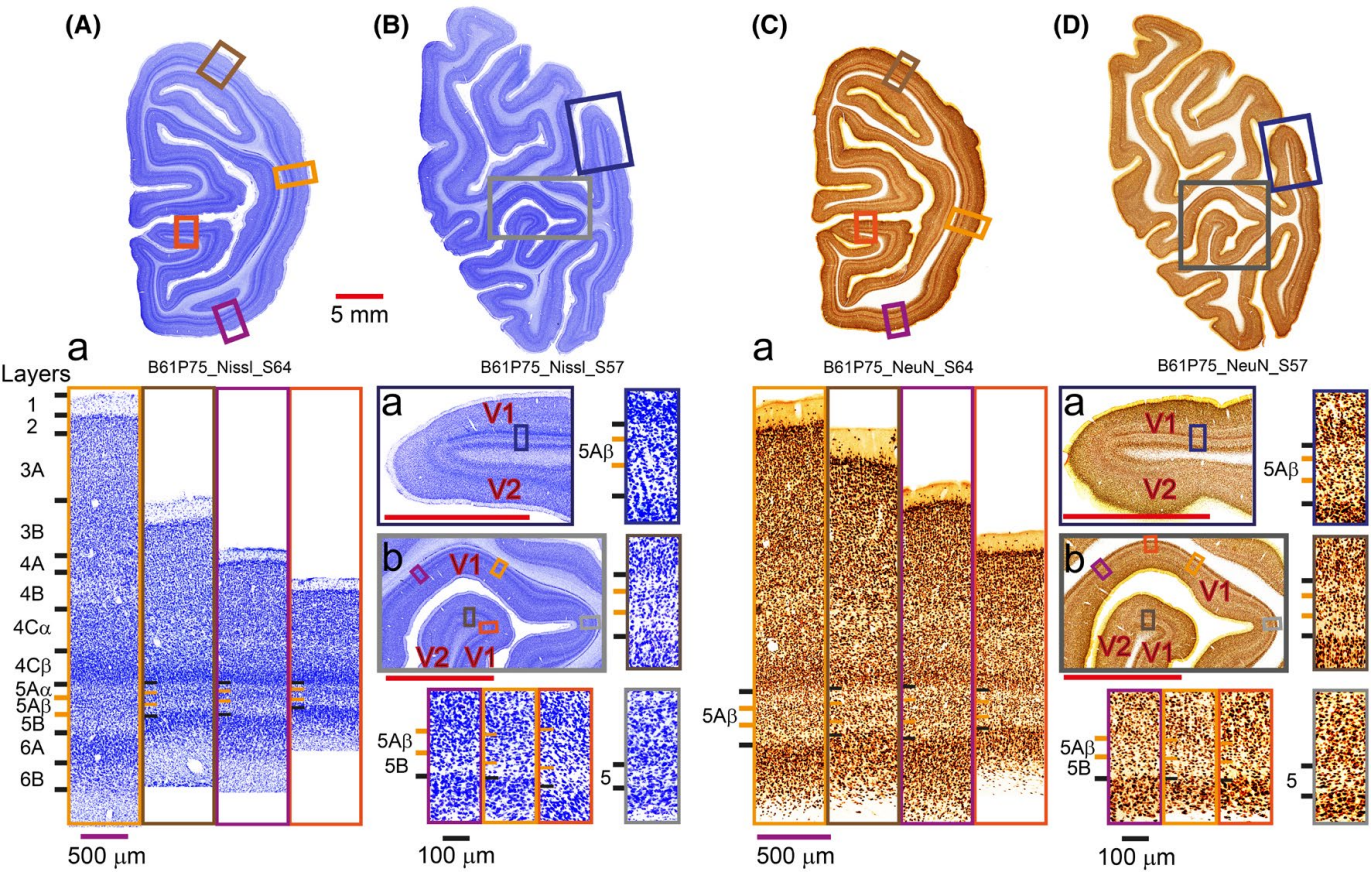
Nissl (A, B) stained and NeuN (C, D) immuno‐labelled coronal sections are from a P75 macaque. (A, C) are coronal sections at the level of the opercular region and to their right (B, D) are coronal sections at the level of the calcarine fissure. Lower figures (A, a and C, a) are tangential sections through the cortex at four locations (denoted by colour‐coded rectangles) showing the trilaminar arrangement of layer 5, denoted as 5Aα, 5Aβ and 5B. (B, a and D, a) are sections around the opercular region and to their right is a single section around layer 5 showing again the trilaminar arrangement. (B, b and D, b) show the cortex around the calcarine region with evidence of a trilaminar layer 5. However, around this region we mainly find layer 5 to be bilaminar (denoted as layers 5Aβ and 5B) as shown in the figures below (B, b and D, b). Again, the cortical location at which the respective tangential sections are shown are denoted by the colour‐coded rectangles. Note that at the sulcal locations, the thickness of layer 5 was usually too small for us to discern a lamination pattern, and is thus denoted as a single lamina (layer 5).

**FIGURE 3:**
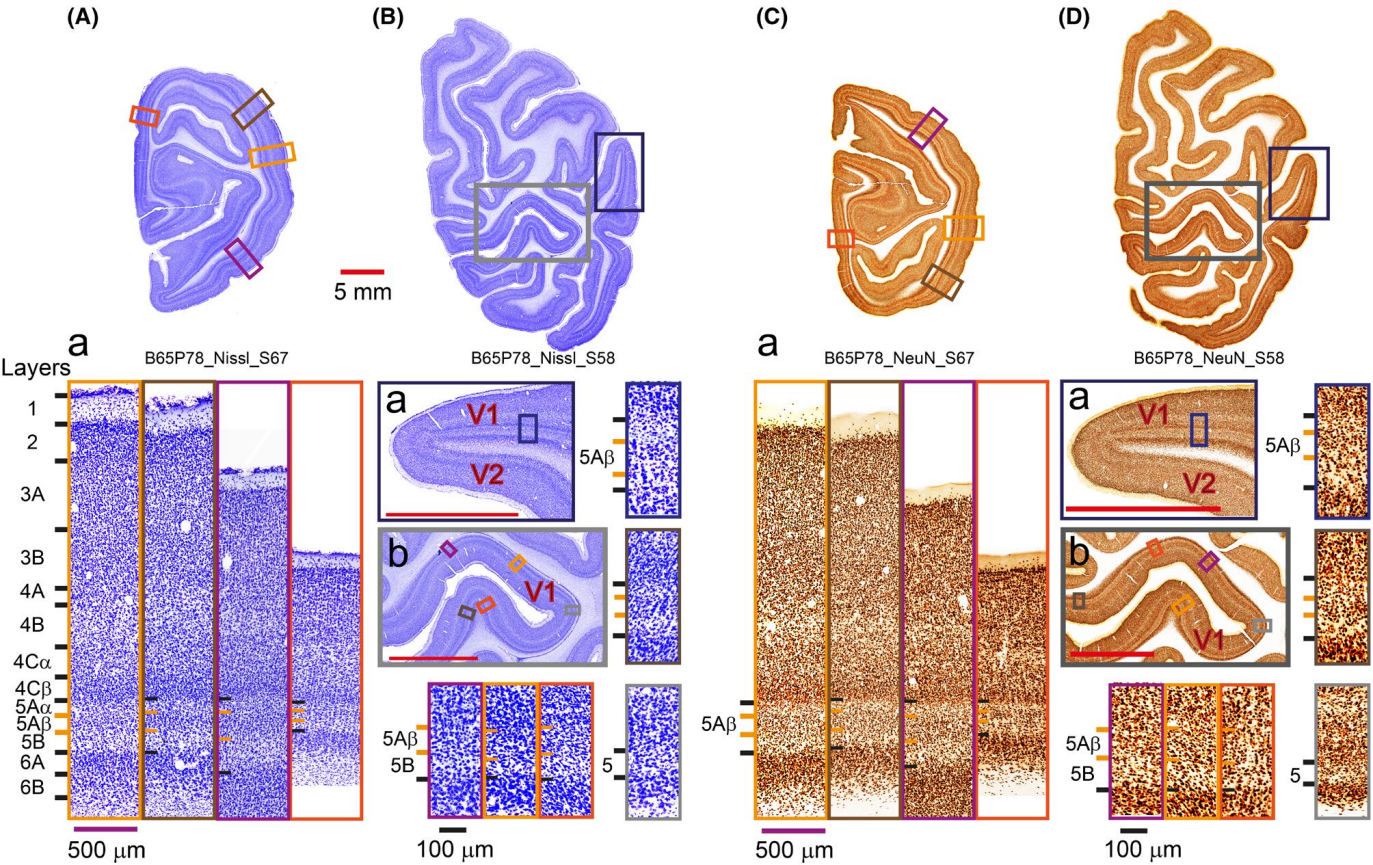
Nissl (A, B) stained and NeuN (C, D) immuno‐labelled coronal sections are from a P78 macaque. Details as in Figure 2.

**FIGURE 4:**
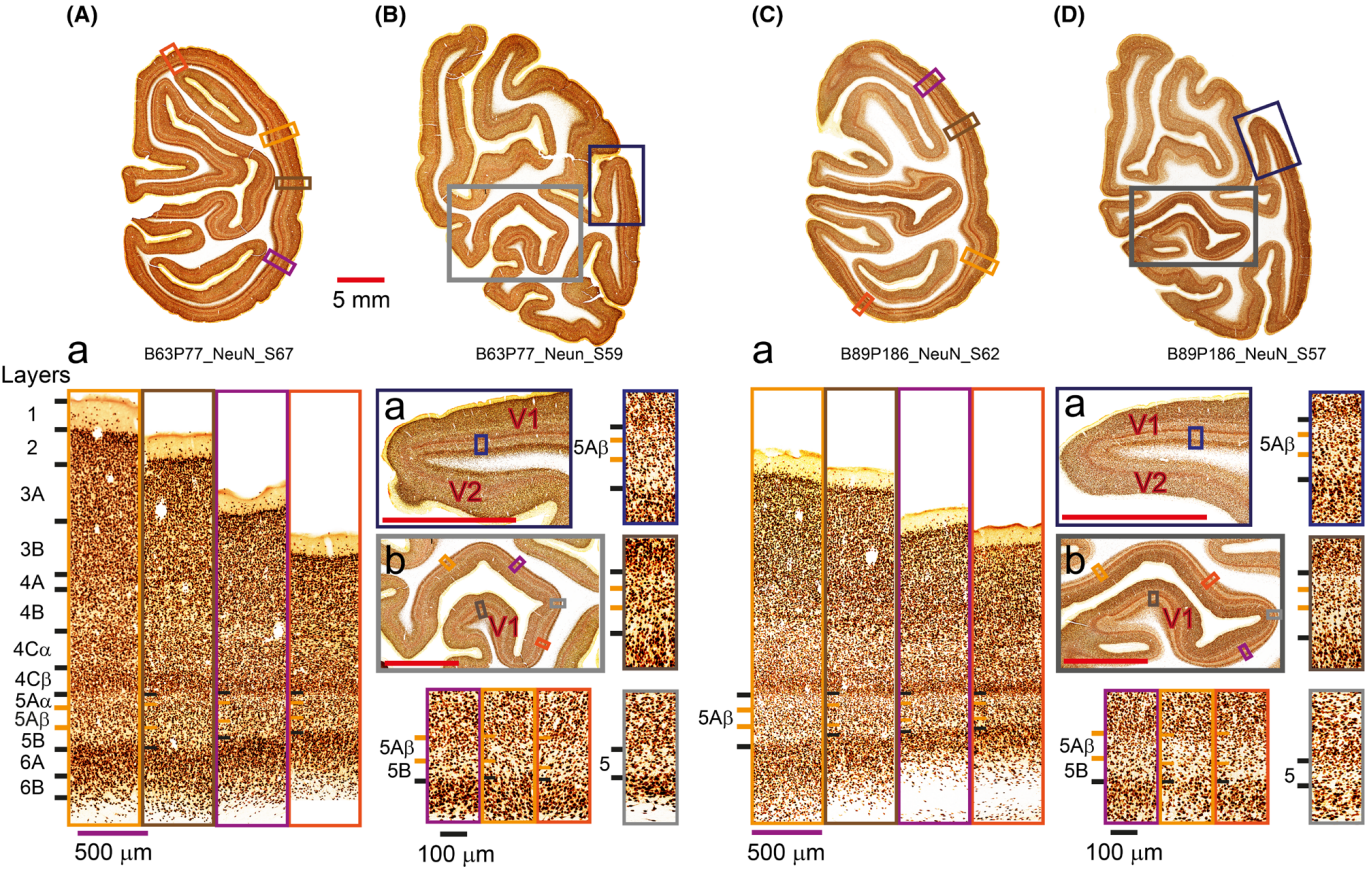
NeuN immuno‐labelled coronal sections are from two macaques (A, B for B63P77) and a six‐month‐old macaque (C, D for B89P186). Details as in Figure 2.

We have examined regions of the primary visual cortex (V1) around the opercular region of the occipital cortex and that around the calcarine fissure in 9 macaques with normal vision and one where the eyes were enucleated in the embryo. We have found layer 5 to be a trilaminar layer, which we have denoted as layers 5Aα, 5Aβ, and 5B. This trilaminar layer arrangement is prominent in both Nissl and NeuN labelled material of V1 over the opercular region, whereas, mostly, a bilaminar layer 5 (denoted as layers 5Aβ and 5B) is found within V1 overlying the calcarine region (Figures 2, 3, 4). A small region within V1 of the calcarine fissure had a trilaminar arrangement of layer 5. This trilaminar arrangement of layer 5 was exclusively found at the gyral location within the calcarine fissure (see Figures 2, 3, 4, Bb, Db). The extent of layer 5Aα in V1 of the calcarine fissure varied between macaques covering a length from 3.4% to 14.8% (mean 8.9% ±0.4%, *n* = 73 sections) of the length of layer 5. The measured mean length of layer 5Aα was 2.8 mm (1 ± SEM = 0.6 mm) across the calcarine fissure which had a layer 5 mean length of 33.4 mm (1 ± SEM = 1.8 mm). Figures 2 and 3, show these arrangement for Nissl‐stained sections (Figures 2A,B and 3A,B) and for NeuN immuno‐labelled sections (Figures 2C,D, 3C,D and 4A–D).

Within the opercular region, layer 5Aβ is a high cell density layer bounded dorsally by a thin sub‐layer of lower density cells (layer 5Aα) and ventrally by sub‐layer 5B. The measured thickness of layer 5Aα was significantly different between Nissl‐stained and NeuN immuno‐labelled sections (mean ± 1 SEM, with Nissl 62.6 ± 3.2 μm, with NeuN 73.7 ± 2.9 μm; *p* < 0.02). The greater thickness of layer 5Aα of NeuN immuno‐labelled compared with Nissl labelled sections at the opercular region of normal vision macaques is probably related to the difficulty of defining the boundaries in the Nissl material between, dorsally, layer 4Cβ and, ventrally, layer 5Aβ. Hence, this lower value could be explained by staining of the non‐neuronal cells by the Nissl stain making it more difficult to see laminar boundaries. There was no significant difference between the thicknesses of layer 5 by these labels (mean ± 1 SEM, with Nissl 315 ± 11.6 μm, with NeuN 318.8 ± 10.2 μm; *p* > 0.08).

**FIGURE 5:**
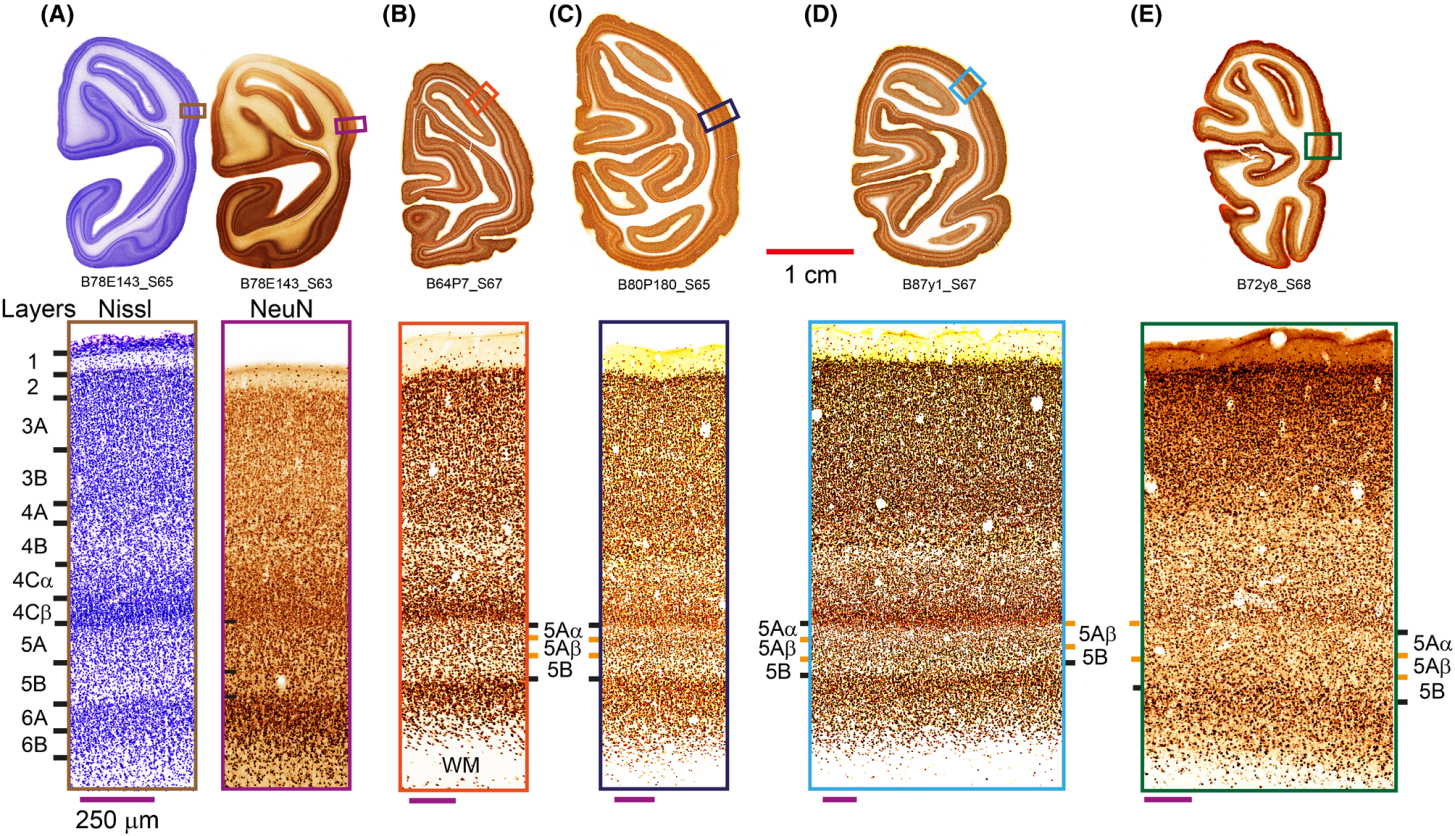
Coronal sections are from the opercular region of V1 of the occipital cortex. (A) are Nissl‐stained and NeuN immuno‐labelled sections at gestational age of 143 days. (B–E) NeuN immuno‐labelled sections at, respectively, ages of 7 days, 6 months, 1 year and 8 years. Below each is the tangential section through the cortex, denoted by the colour‐coded rectangles, at the locations on the coronal sections. Layer 5 lamination becomes prominent from age 7 days as a trilaminar layer. With age, layers 5Aα and 5Aβ become patchy such that lamina 5Aα becomes less prominent, giving rise to a bilaminar layer 5, denoted by 5Aβ and 5B.

**FIGURE 6:**
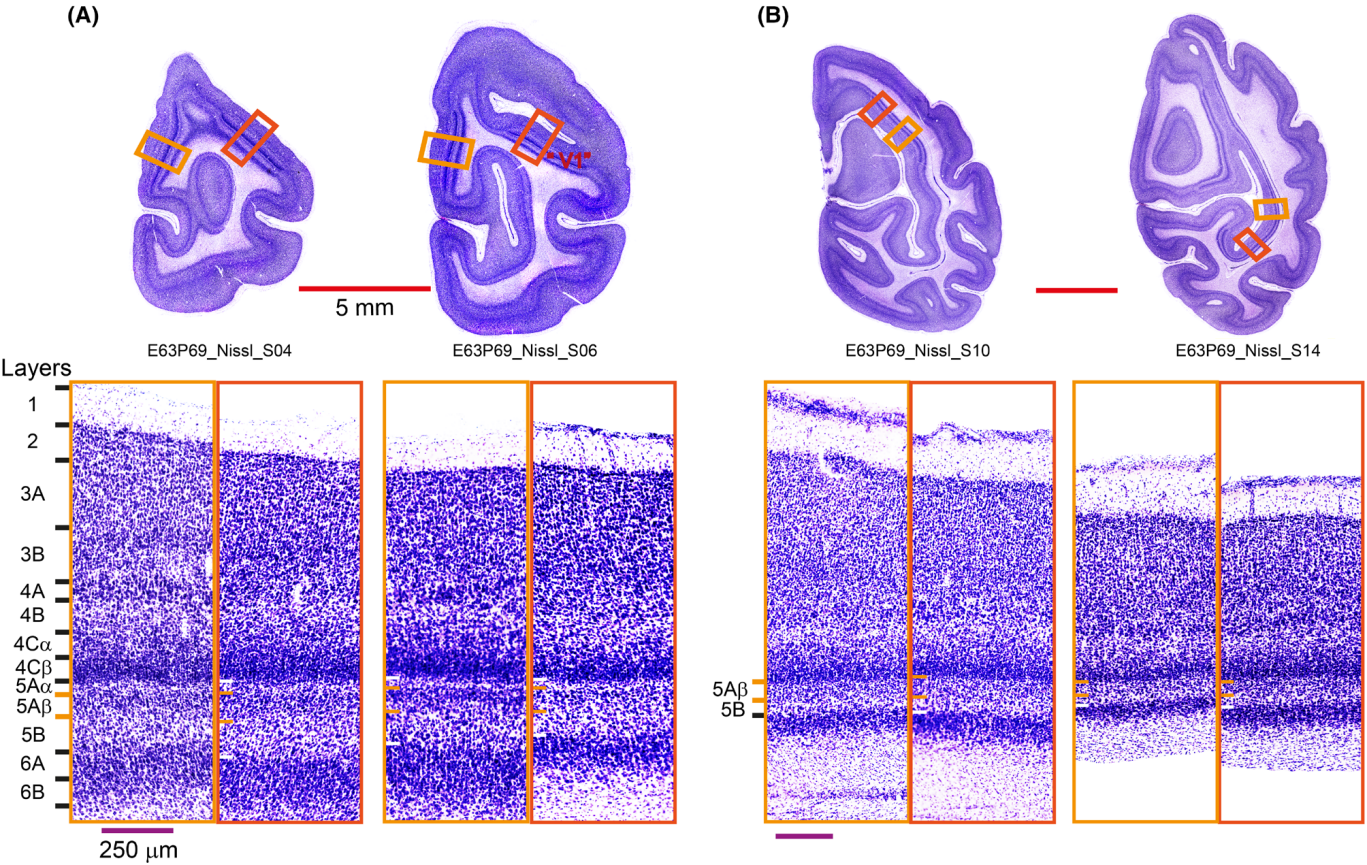
Nissl‐stained coronal sections shown are from the enucleated macaque. (A) shows sections through the caudal region of the occipital cortex and (B) from a more anterior region around the calcarine region. Below these sections, we show tangential sections through the cortex at regions of cortex displaying V1‐like lamination (denoted by colour‐coded rectangles). Layer 5 has a prominent trilaminar structure in (A) and a bilaminar in (B).

Layers 1–6 are known to vary in thickness across different regions of cortex (see Figures 2, 3, 4, 5, 6; Ahmed et al., 2024). The thicknesses of the layers across V1 presumably scale with cortical thickness. Our measurements of V1 show that at the opercular regions V1 was significantly thicker than at the calcarine regions (mean ± 1 SEM, respectively, 2271.6 ± 58.8 μm; 1644.1 ± 29.9 μm; *p* < 0.01). This was also true for layer 5 (mean ± 1 SEM, respectively, 316.9 ± 7.7 μm; 191.1 ± 7.6 μm; *p* < 0.01).

We examined coronal sections from the opercular region of macaques at various ages. For an embryonic Macaque of gestational age 143 days (B78E143), Nissl labelled and NeuN immuno‐labelled coronal sections show layer 5 of V1 to be bilaminar (Figure 5A). At this gestational age layer 5 is bilaminar and we find a trilaminar layer 5 at a postnatal age of 7 days (B64P7, Figure 5B). The evidence we have, therefore, is that layer 5 of the embryonic brain up to 143 days of gestational age remains bilaminar. The trilaminar arrangement of layer 5 is present at postnatal age of 7 days and becomes prominent within the first year after birth. However, with older macaques the trilaminar arrangement becomes irregular. The thin layer 5Aα occurs intermingled with a bilaminar arrangement of layers 5Aβ and 5B, where layer 5Aβ juxtaposes dorsally to layer 4Cβ and ventrally to layer 5B (Figure 5).

We analysed layer 5 of V1 of a bilaterally enucleated (embryonic age of 63 days) macaque at a postnatal age of 69 days. In this macaque, layer 5 was found to be trilaminar around the occipital cortex and bilaminar around the calcarine region (Figure 6). In this macaque, we found V1 like lamination pattern interdigitated amongst extra‐striate cortex, with lamination similar to secondary visual area (V2), over the occipital cortex and the calcarine region. Our measurements of V1 cortical thicknesses at occipital and calcarine region contrasted with macaques of normal visual experience in that the thicker V1 was found in the calcarine region (mean ± 1 SEM, respectively, 1172.1 ± 30.5 μm; 1282.6 ± 44.9 μm; *p* < 0.03). The thickness of layer 5Aα was 34.6 ± 1.3 μm (mean ± 1 SEM) whereas in macaques age matched (*n* = 3), with normal visual experience, the layer is thicker (76.1 ± 5.5 μm, *p* < 0.01). Table 1 is a summary of the measurements of V1 cortical thickness as well as of layer 5.

**TABLE 1:** Averaged measured thicknesses of cortex, layer 5, sublayers 5Aα and 5Aβ in 8 macaques and the enucleated case. Mean ± 1 SEM.

Thickness (μm)	Normal vision macaques				Enucleated macaque	
	Opercular region		Calcarine region		Opercular region	Calcarine region
	Nissl	NeuN	Nissl	NeuN	Nissl	Nissl
Cortical	2260.8 ± 87	2282.3 ± 80.2	1653.3 ± 44.8	1634.9 ± 40.4	1172.1 ± 30.5	1282.6 ± 44.9
Layer 5	315.1 ± 11.6	318.8 ± 10.2	193.8 ± 9.8	188.3 ± 9.4	198.8 ± 6.6	142.8 ± 7.3
Layer 5Aα	62.6 ± 3.2	73.7 ± 2.9	54.1 ± 3.4	52.4 ± 4.8	34.6 ± 1.3	
Layer 5Aβ	104.8 ± 5.0	114.0 ± 4.4	86.1 ± 4.6	78.9 ± 4.5	63.6 ± 2.5	68.1 ± 3.2

The values in Table 1 for macaques with normal visual experience reveal that cortical thickness of V1 differs significantly between the opercular and calcarine regions (*p* < 0.01). This is also true for the sublayers of layer 5 (*p* < 0.01).

For the enucleated case, the cortical thickness of V1 at the calcarine region was significantly greater than at the opercular region (*p* < 0.03), and with the absence of a layer 5Aα at the calcarine region, layer 5 at the opercular region was thicker (*p* < 0.01).

## 4 DISCUSSION

We have analysed the lamination pattern of primary visual cortex (V1) in two regions of cortex – the opercular region of the occipital cortex and cortex overlying the calcarine fissure. Our main findings are that layer 5 has three sub‐laminae in the opercular region and mainly two sub‐laminae in the calcarine region. In addition, in a bilaterally enucleated macaque at an embryonic age of 63 days, at a postnatal age of 69 days, this macaque had a similar lamination pattern to macaques with normal visual development (Figure S1). This supports the concept that the geniculo‐cortical input and the developing occipital and calcarine regions do not require visual experience to form the unique laminated structure of V1 (Figures 2, 3, 4, 6) (Bourgeois & Rakic, 1996; Horton & Hocking, 1996; Karlen & Krubitzer, 2009; Rakic, 1977, 1988; Rakic et al., 1991).

Layer 5 has been historically subdivided into two sublayers, 5A and 5B (Lund, 1987, 1988; Lund & Boothe, 1975). Layer 5A is a thin layer, ~50–100 μm in thickness, lying ventral to layer 4Cβ, and layer 5B dorsal to layer 6A. Our findings of layer 5, as a trilaminar structure, have a high density of neuronal cells within layer 5Aβ which is sandwiched between a lower cell density layer 5Aα dorsally, and a sub‐layer we now denote as layer 5B ventrally. Layer 5Aα is equivalent to layer 5A of Lund and Boothe (1975) and in agreement with them we find the mean thickness of this layer to be 68.0 μm (*N* = 8, locations = 77, 1 SEM = ±2.25 μm). However, we show that this layer develops with age and that in older macaques (> ~1 year of age) this layer occurs in patches such that layer 5Aβ intermittently occupies this zone (Figure 5). In addition, we find that layer 5Aα is normally absent from V1 overlying the calcarine region. We have divided the historical layer 5B into layers 5Aβ and 5B and not layers 5Bα and 5Bβ. Our reasoning behind this is that outside the opercular striate cortex region, layer 5 is bilaminar not only across visual extra‐striate cortex V2 but across other cortical areas. At these areas, layer 5A can be distinguished from layer 5B by possessing a higher cell density (Figures S2 and S3). These sublayers can be distinguished by differences in cell densities in Nissl and NeuN labelled material (Figures 2, 3, 4).

Our measurements of tangential cortical thickness have shown that V1 differs significantly between the calcarine region where it is substantially thinner (72.4%; *p* < 0.01) than in the opercular region. Similarly, layer 5 thickness is also reduced (60.3%, *p* < 0.01). These differences may be a consequence of visual field representations, as the opercular region is responsible for our foveal and parafoveal vision (Daniel & Whitteridge, 1961; Essen & Zeki, 1978; Zeki, 1969).

The analyses of V1 like zones in the enucleated macaque have followed the same pattern as those with normal visual experience. It has been well established that in bilaterally enucleated macaques during the prenatal period, postnatally their opercular region of the occipital cortex develops folds (sulci) unlike for normal macaques where this region is smooth, their V1 like lamination pattern interdigitates with a lamination pattern similar to extra‐striate cortex (V2) (Dehay et al., 1996; Rakic et al., 1991), and within the folded opercular region zones of hybrid cortex are formed (Rakic et al., 1991). However, in this enucleated macaque two major differences were evident. Firstly, layer 5Aα was much thinner being 34.6 μm, a reduction to 45.5% of normal layer 5Aα thickness. Secondly, the tangential thickness of V1 zones was also substantially reduced in size. In the posterior occipital cortex, the cortical thickness was reduced to 47.8% and in the calcarine region to 73.1%. It seems that although, V1 like lamination pattern is apparent within these zones, there must be substantial reorganisation due either to cell density changes within the cortical layers or externally mediated by afferents or both (Bourgeois & Rakic, 1996; Horton & Hocking, 1996; Karlen & Krubitzer, 2009; Rakic, 1977, 1988; Rakic et al., 1991).

In conclusion, we show histological differences between the opercular and calcarine regions of the primary visual cortex. This is not unexpected. The opercular region is involved in foveal and central visual field analyses. The region, functionally, has fine receptive fields resulting in high visual acuity (Daniel & Whitteridge, 1961; Parker & Hawken, 1985; Sarmiento, 1975; Tootell, Switkes, et al., 1988), has fine binocular disparities resulting in stereoscopic vision and acute depth perception (Bough, 1970; Cowey et al., 1975; Ohzawa et al., 1990; Poggio & Fischer, 1977), and has exquisite colour and hue perception (Dow & Gouras, 1973; Gouras, 1974; Hanazawa et al., 2000; Lennie et al., 1990; Livingstone & Hubel, 1988; Michael, 1981; Tootell, Silverman, et al., 1988; Xiao et al., 2007). These are among many other substantive adaptations for visual analyses between central and peripheral visual fields (DeValois et al., 1982). Although both these regions receive geniculate input, it is likely that the details of their internal and external anatomical connections vary.

## CONFLICT OF INTEREST STATEMENT

The authors declare no conflict of interest.

## Supporting information


Figure S1.

Figure S2.

Figure S3.

Appendix S1.

## Data Availability

The data that support the findings of this study are available on request from the corresponding author. The data are not publicly available due to privacy or ethical restrictions.
