# Supplementary material for: Lamination of primary visual cortex in the macaque: Layer 5 subdivisions
Source: J Anat. 2026 Apr 9:10.1111/joa.70137. Online ahead of print. doi: 10.1111/joa.70137 (PMC13399498; doi:10.1111/joa.70137)
Supplement: Supplementary file 4 — Appendix S1. [file JOA-9999-0-s002.docx]

Figure S1 NeuN immuno-labelled sections (A, B) are from a 143 day embryonic macaque and Nissl-labelled sections (C, D) are from a binocularly enucleated macaque at embryonic age of 63 days and sacrificed at postnatal age of 69 days. Sections A, C are at the level of the lateral geniculate nucleus (LGN) and to their right is the inset of the LGN. Below A and C are a tangential section through the LGN with its layers denoted by P (parvocelluar), M (magnocellular), K (Koniocellular), and contralateral (c) and ipsilateral (i). The LGN of section C showed no clear laminations. Sections B and D are at the occipital cortex (in B approximately 7 mm, and D ~ 4.5 mm anterior from the occipital pole). Below are tangential sections (taken at locations from B, D). For section B, at the opercular and calcarine regions of primary visual cortex (V1) with V1 like lamination pattern but at this age layer 5 is a bilayer. For section D, a short length of V1 like lamination pattern interdigitates with an extra-striate lamination pattern. Within the occipital region, layer 5 was trilaminar (denoted layers 5Aα, 5Aβ, and 5B) and within the calcarine region it was bilaminar (layers 5A, 5B). WM – white matter.

Figure S2 NeuN immuno-labelled sections (A–D) are from two 6-month-old macaques (B80P180 and B89P186). Sections A and C are from the occipital cortex (A, C ~ 16 mm anterior from the occipital pole). The opercular regions of V1 in A and C, have a layer 5 which is trilaminar (denoted V1). Outside V1 in these sections layer 5 is bilaminar (two examples are shown below sections A, C). Sections B, D are at the level of the lateral geniculate nucleus (LGN). Below B and D sections, we show the cortical layering pattern in four locations. These were chosen based on a discernable tangential lamination pattern from the pial surface to the white matter. The layering confirms that layer 5 in these regions is a bilaminar.

Figure S3 NeuN immuno-labelled sections (A, C) and Nissl-stained sections (B. D) are from macaque B65P78. Below A and B are tangential sections from the opercular cortical region denoting the trilaminar arrangement of layer 5, and below C and D are sections from the calcarine region displaying the bilaminar arrangement of layer 5.
