# Supplementary material for: Lamination of primary visual cortex in the macaque: Layer 5 subdivisions
Source: J Anat. 2026 Apr 9:10.1111/joa.70137. Online ahead of print. doi: 10.1111/joa.70137 (PMC13399498; doi:10.1111/joa.70137)
Supplement: Supplementary file 1 — Figure S1. [file JOA-9999-0-s004.pdf]

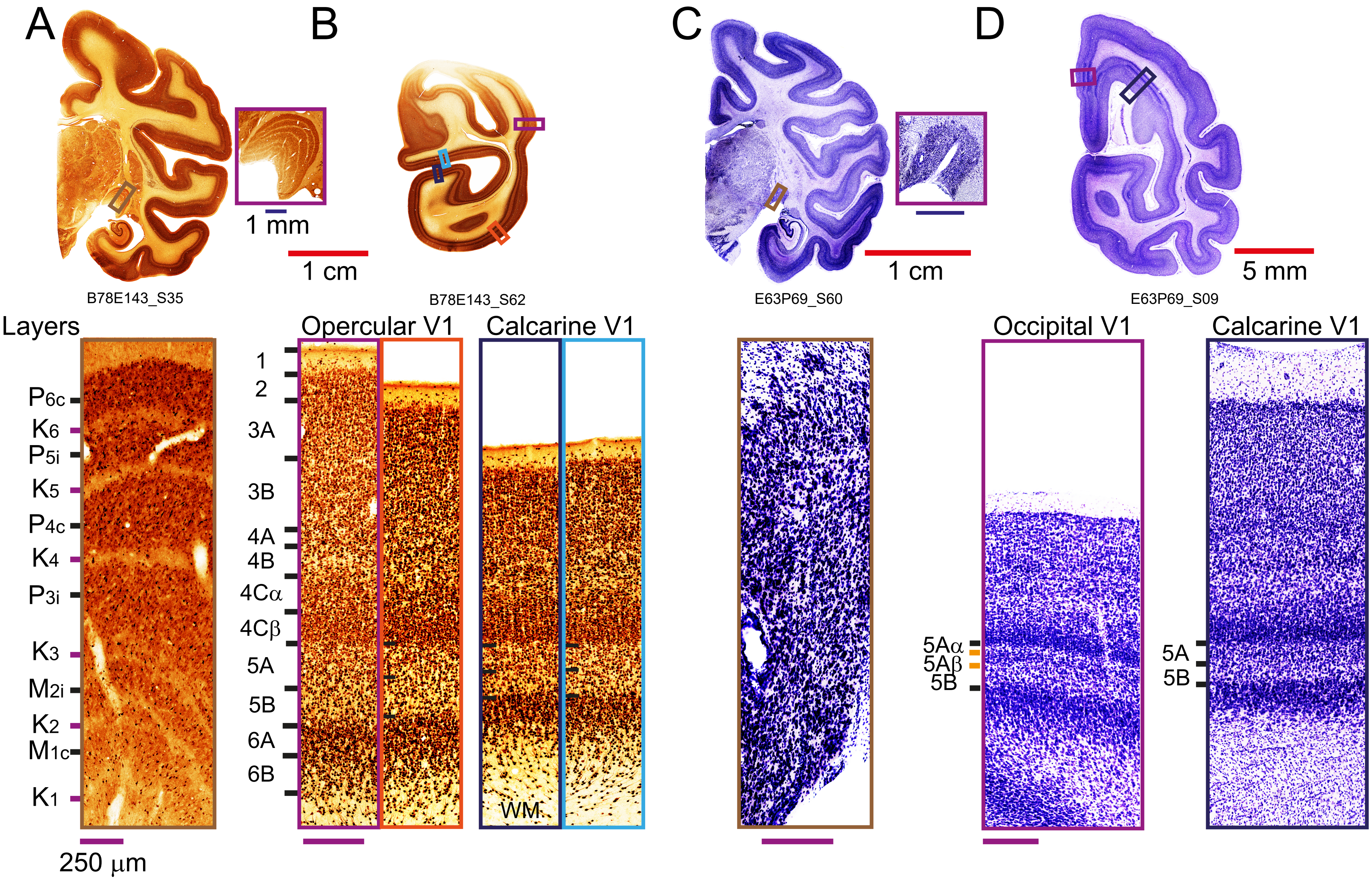

A
B
C
D
1 mm
1 cm
1 cm
5 mm
B78E143_S35
B78E143_S62
E63P69_S60
E63P69_S09
Layers
Opercular V1
Calcarine V1
Occipital V1
Calcarine V1
P6c
K6
P5i
K5
P4c
K4
P3i
K3
M2i
K2
M1c
K1
1
2
3A
3B
4A
4B
4Cα
4Cβ
5A
5B
6A
6B
WM
5Aα
5Aβ
5B
5A
5B
250 µm
