# Supplementary material for: Lamination of primary visual cortex in the macaque: Layer 5 subdivisions
Source: J Anat. 2026 Apr 9:10.1111/joa.70137. Online ahead of print. doi: 10.1111/joa.70137 (PMC13399498; doi:10.1111/joa.70137)
Supplement: Supplementary file 2 — Figure S2. [file JOA-9999-0-s001.pdf]

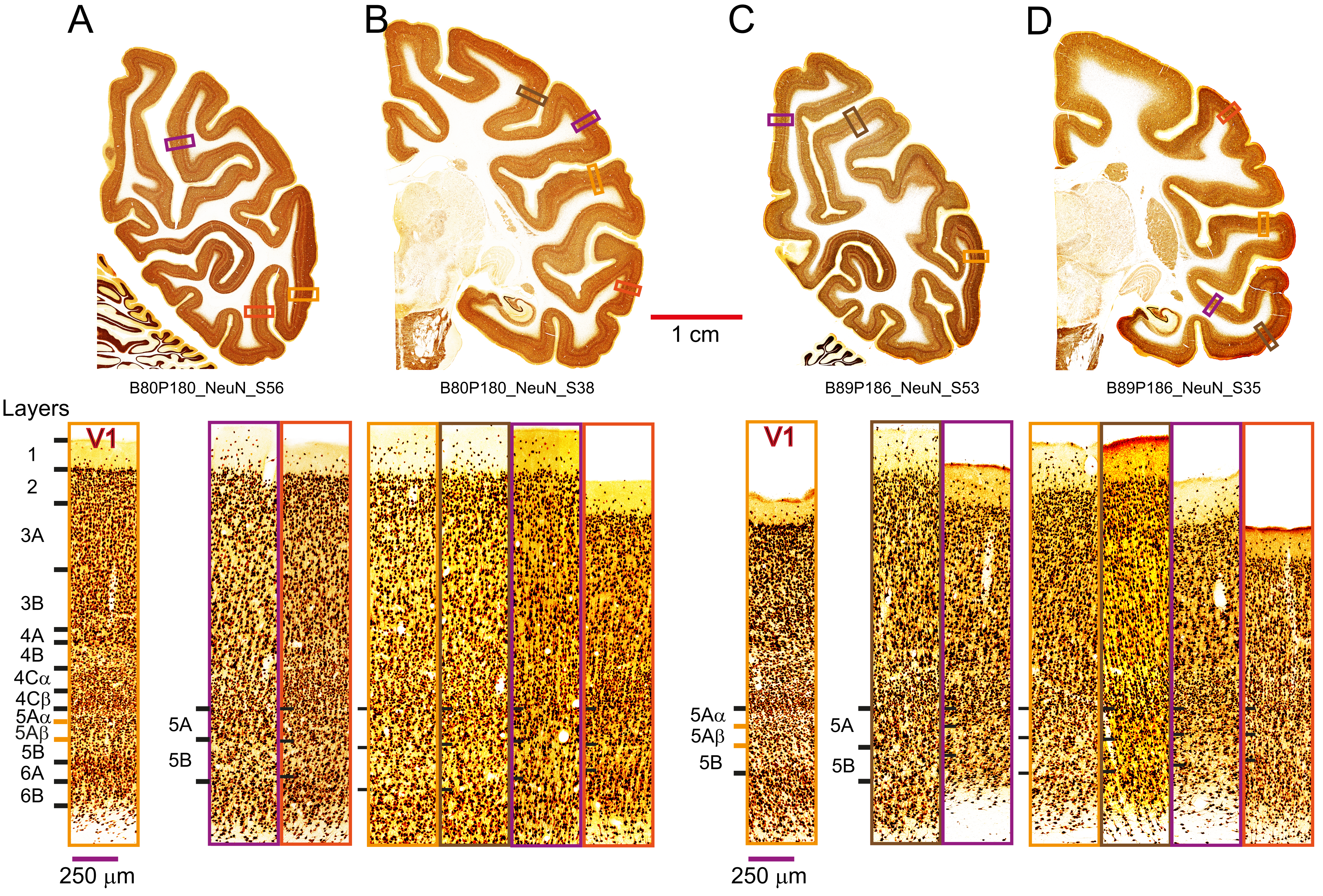
A
B
C
D
1 cm
B80P180_NeuN_S56
B80P180_NeuN_S38
B89P186_NeuN_S53
B89P186_NeuN_S35
Layers
V1
1
2
3A
3B
4A
4B
4Cα
4Cβ
5Aα
5Aβ
5B
6A
6B
5A
5B
V1
5Aα
5Aβ
5B
5A
5B
250 μm
250 μm
