# Supplementary material for: Lamination of primary visual cortex in the macaque: Layer 5 subdivisions
Source: J Anat. 2026 Apr 9:10.1111/joa.70137. Online ahead of print. doi: 10.1111/joa.70137 (PMC13399498; doi:10.1111/joa.70137)
Supplement: Supplementary file 3 — Figure S3. [file JOA-9999-0-s003.pdf]

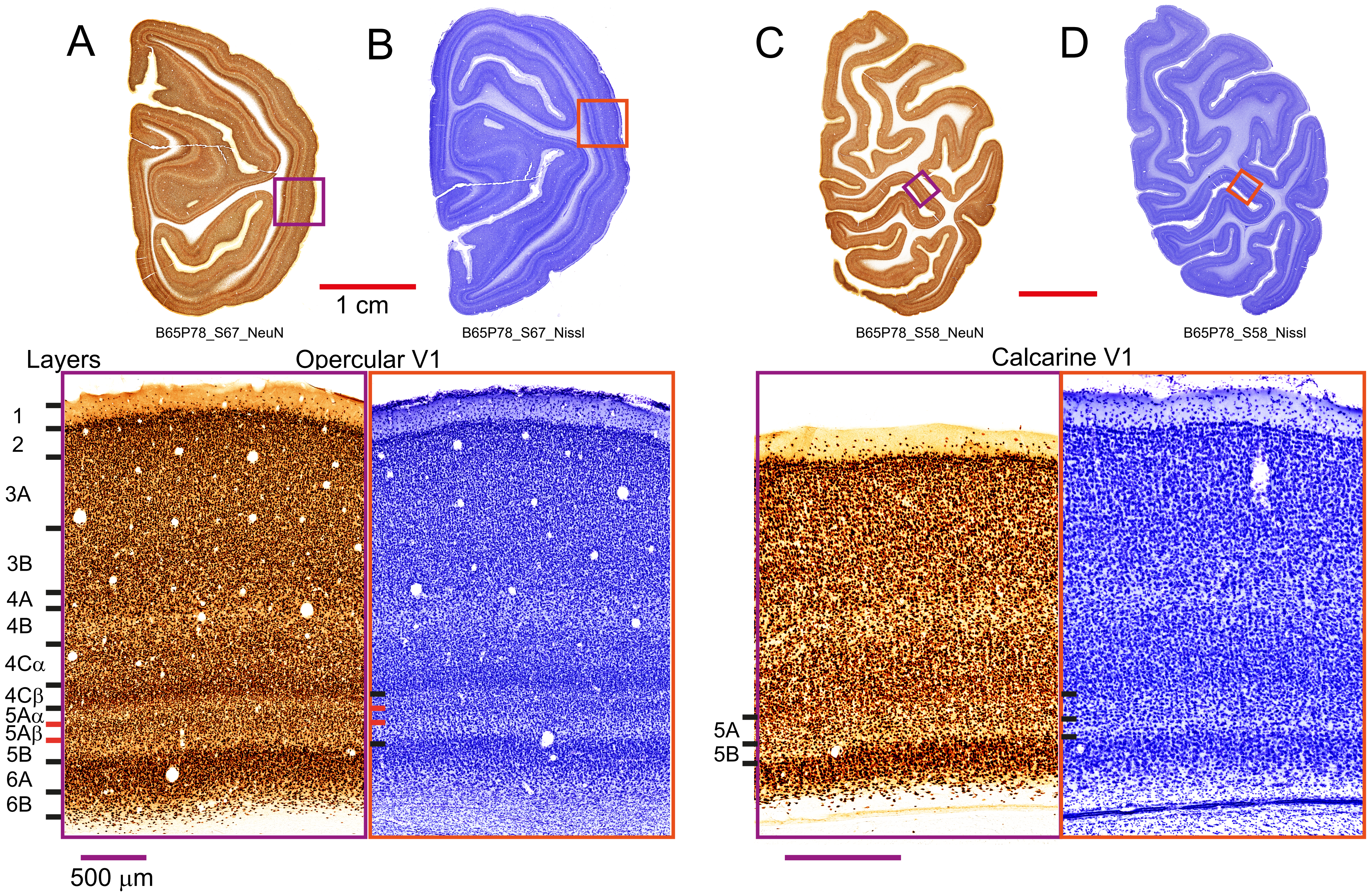

A
B
C
D
1 cm
B65P78_S67_NeuN
B65P78_S67_Nissl
B65P78_S58_NeuN
B65P78_S58_Nissl
Layers
Opercular V1
Calcarine V1
1
2
3A
3B
4A
4B
4Cα
4Cβ
5Aα
5Aβ
5B
6A
6B
5A
5B
500 μm
